# Cement augmentation of bone defect in pathological humeral diaphyseal fracture treated with retrograde intramedullary nail

**DOI:** 10.1308/003588412X13373405386015l

**Published:** 2012-09

**Authors:** J Dhaliwal, A Seif, S Singh, A Sinha

**Affiliations:** Betsi Cadwaladr University Health Board,UK

## BACKGROUND

**Figure 1:**
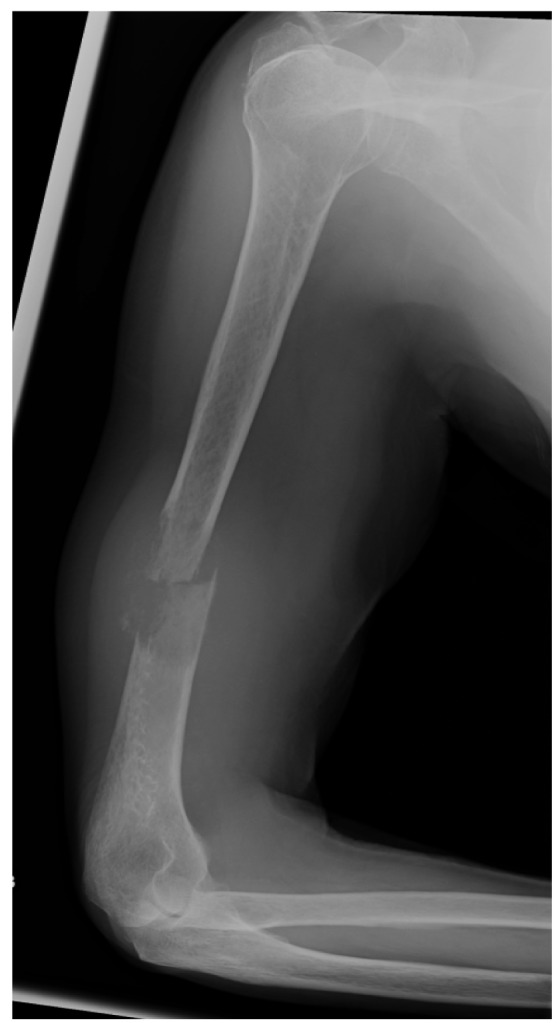
Lytic lesion in right humerus

**Figure 2:**
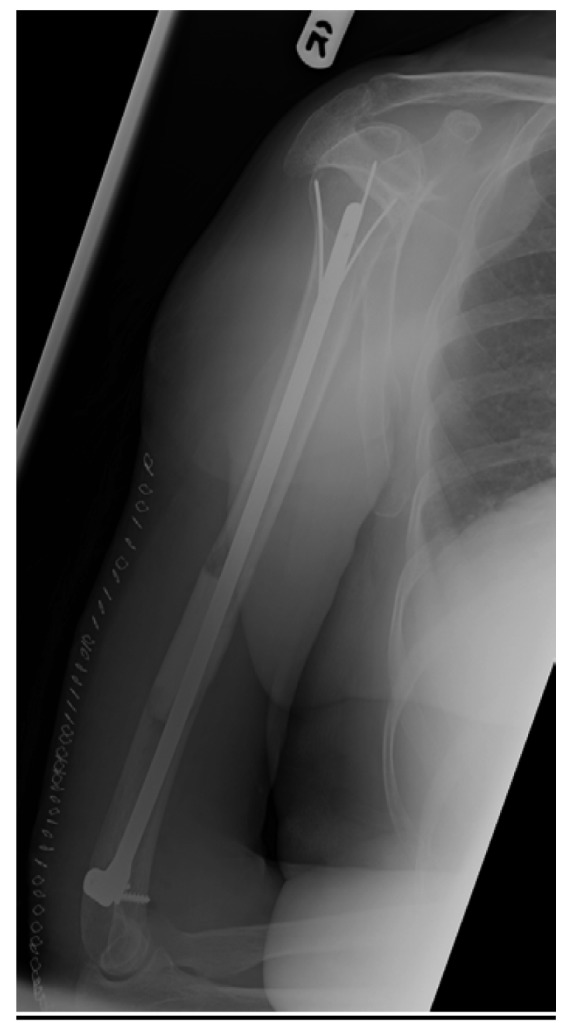
Post-operative right humerus

Pathological fractures of long bones secondary to metastatic lesions are common.^1^ Management of pathological humeral diaphyseal fractures with bone loss is challenging. Palliative internal fixation improves pain and quality of life.^2^ We describe a simple technique to address bone loss of the distal humerus (Fig 1) using Palacos® bone cement (Heraeus, Wehrheim, Germany) following retrograde humeral internal fixation (Fig 2).

## TECHNIQUE

**Figure 3:**
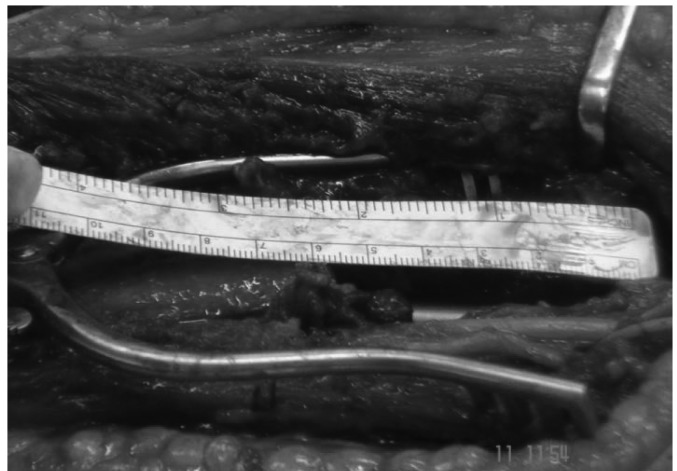
5cm bone defect in distal humerus

The patient was placed in a lateral position with the operative limb over an armrest. The posterior approach was taken to the elbow with an incision through the triceps aponeurosis down to the bone. A tumour involving soft tissue was debulked and curettage of the lesion performed. There was a 6cm bone defect involving the distal third of the humerus (Fig 3). The fracture was reduced and a retrograde Halder intramedullary nail (7mm x 270mm) introduced with distal locking screws.

**Figure 4:**
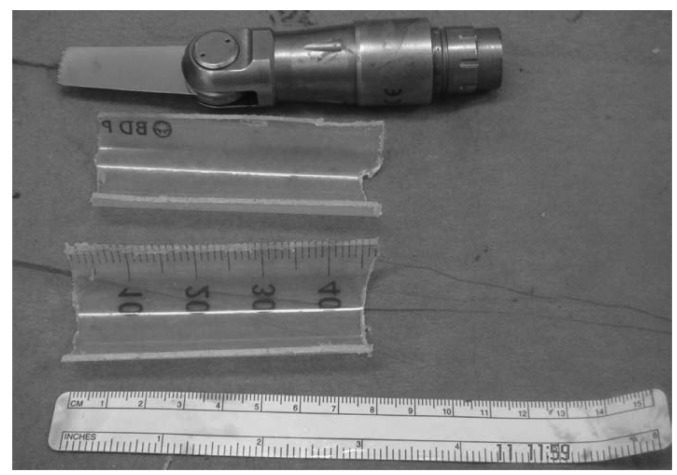
50ml syringe divided sagittally to relevant size

**Figure 5:**
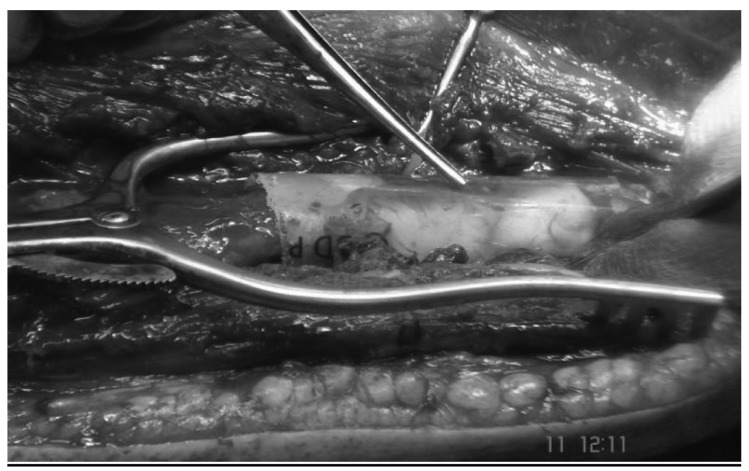
Intramedullary nail and cement enclosed by two halves of syringe

**Figure 6:**
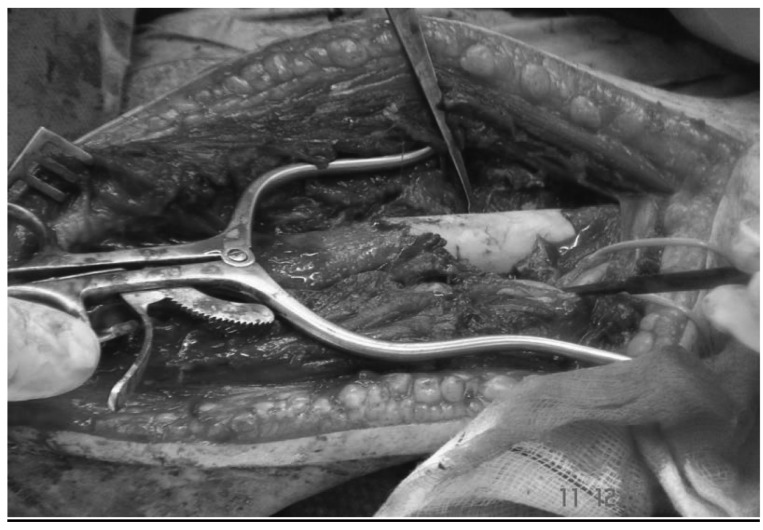
A tube of smooth set cement surrounds intramedullary nail and bridges bone defect

A 50ml syringe was cut using a saw to prepare a mould (Fig 4). One half of the syringe was placed posteriorly to the nail and prepared Palacos® cement was placed in the syringe and around the nail. The other half of the syringe was placed anteriorly to encase the nail and cement. The plastic syringe mould was removed on setting of the cement (Fig 5). The cement mantle bridged the entire bone defect (Fig 6). This was followed by closure in layers.

## DISCUSSION

This technique enables a tube of smooth cement mantle to be created, surrounding the intramedullary nail at the site of the bone defect. The equipment and material necessary are readily available in most operating theatres.
